# The levels of urinary glycosaminoglycans of patients with attenuated and severe type of mucopolysaccharidosis II determined by liquid chromatography-tandem mass spectrometry

**DOI:** 10.1016/j.ymgmr.2016.03.009

**Published:** 2016-04-22

**Authors:** Ryuichi Mashima, Eri Sakai, Misa Tanaka, Motomichi Kosuga, Torayuki Okuyama

**Affiliations:** a Department of Clinical Laboratory Medicine, National Center for Child Health and Development, 2-10-1 Okura, Setagaya-ku, Tokyo 157-8535, Japan; b Division of Medical Genetics, National Center for Child Health and Development, 2-10-1 Okura, Setagaya-ku, Tokyo 157-8535, Japan; c Center for the Lysosomal Storage Disorder, National Center for Child Health and Development, 2-10-1 Okura, Setagaya-ku, Tokyo 157-8535, Japan

**Keywords:** Mucopolysaccharidosis, Glycosaminoglycans, LC-MS/MS, Hunter syndrome

## Abstract

Glycosaminoglycans (GAGs) play important roles on the regulation of extracellular signaling, neuronal development, and cartilage maintenance. The extracellular concentration of total GAGs has been used as an established measure for the diagnosis of mucopolysaccharidoses (MPSs). Heparan sulfate (HS), Dermatan sulfate (DS) and chondroitin sulfate are known to be elevated in the GAGs under pathological conditions associated with MPS. Furthermore, the selective accumulation of disease-specific one of, or a combination of, them has also been used for the estimation of subtypes of MPS. A previously developed method [Auray-Blais C et al. Molecular Genetics and Metabolism 102 (2011) 49–56.] measures the concentration of GAGs using liquid chromatography with tandem mass spectrometry (LC-MS/MS) with higher precision. To ask whether the selective accumulation of HS and DS in the urine of MPS II patients discriminate the attenuated and severe type of MPS II, we examined the concentrations of HS and DS by this methodology. Compared to the healthy controls, we found a marked elevation of HS and DS in all of the MPS II-affected patients. Among patients who received ERT with confirmed elevation of antibody titer, the concentrations of HS in the urine of patients with attenuated type were lower than those with severe type of MPS II. In these patients, the concentrations of DS by LC-MS/MS and of total GAG by DMB failed to depend on the accumulation of antibody. These results suggest that the LC-MS/MS method employed in this study might discriminate the subtypes of MPS II in different clinical background.

## 1 Introduction

Mucopolysaccharidoses (MPSs) are lysosomal storage disorders (LSDs) characterized by inherited deficiencies of lysosomal enzymes essential for the degradation of mucopolysaccharides [1]. A failure of this metabolism induces selective accumulation of glycosaminoglycans (GAGs) in the blood, urine, and body tissues. Multiple enzymes are involved in clinical presentations of MPS subtypes, thus a defect or significant impairment of the responsible enzyme activity leads to the accumulation of selective GAGs including heparan sulfate (HS), dermatan sulfate (DS) and chondroitin sulfate (CS). Among all MPS subtypes, only MPS II is an X-linked disorder, while the others are autosomal recessive. The clinical manifestation of MPSs involves skeletal deformities, progressive coarse facial features, and organomegaly with mental retardation in some MPS subtypes. The diagnosis based on clinical presentation is normally inconclusive for all MPS subtypes; thus, biochemical and/or genetic confirmatory examination are always required. Among various assays, biochemical measurement of enzyme activity has been accepted as the most promising measure, because some MPS subtypes such as MPS III and IV are caused by four and two distinct enzymes, respectively.

Several therapeutic strategies for MPSs are currently available. The most widely used treatment is enzyme replacement therapy (ERT), which supplies the deficient enzyme intravenously [2], [3]. This therapy improves most systemic manifestation, whereas this is known to be ineffective for intellectual decline and bone destruction under current formulation. To overcome this, a recent study indicated the efficacy of intrathecal administration of iduronate-2-sulfatase (IDS) enzyme due to the significant attenuation of accumulating GAGs in the cerebrospinal fluids (CSFs) [4]. Additionally, a recombinant IDS enzyme that is fused to the fragment of antibody against human insulin receptor has been developed [5]. This strategy aims to facilitate the penetration of IDS enzyme through blood brain barrier via the receptor. In addition to these renovated enzyme agents, hematopoietic stem cell transplantation has also been performed [6], [7]. A recent study demonstrated that the levels of total GAGs in the CSFs discriminates the patients of MPS II with and without cognitive impairment [8].

For diagnostic purposes, the levels of GAGs in clinical specimens have been determined by several methods. One procedure involves alkolytic pretreatment of GAGs followed by the measurement of concentrations of GAG-derived disaccharides using LC-MS/MS [9], [10], [11], [12], [13], [14]. Based on this technique, the effectiveness of ERT for MPS II has been clearly shown in the several studies [10], [11], [14]. Similarly, the detection of a trace amount of HS in the CSF of MPS I-affected individuals has also been reported [9]. Thus, we hypothesized whether the disease subtypes of MPS, such as the attenuated and severe type of MPS II, could be identified using this LC-MS/MS assay.

## 2 Experimental procedure

### 2.1 Reagents

HS, DS, CS and methanolic 3N-HCl were obtained from Sigma Aldrich (St Louis, MO). Acetonitrile and methanol were purchased from Fischer Scientific (Tokyo, Japan). Isopropanol was purchased from Wako Pure Chemicals (Tokyo, Japan). Deionized water was obtained from a Milli-Q water system (Millipore, Milford, MA). Ammonium acetate and formic acid were purchased from Kanto Chemical (Tokyo, Japan). The other reagents used in this study were of the highest grade commercially available.

### 2.2 Patients

After informed consent was obtained, urine samples were collected from MPS patients whose diagnosis was confirmed by the detection of either defective or significantly impaired enzyme activity and/or by mutation analysis. We examined the urinary specimens collected from 8 MPS II patients (4 patients of attenuated type and 4 patients of severe type, respectively) and 4 healthy controls. All of the patients with MPS II have been received ERT. Ages of the patients of attenuated and severe type of MPS II as well as those of the healthy controls at sample collection were 10–40, 10–17, and 8–43, respectively. The mean ages of ERT initiation for the patients with attenuated and severe type of MPS II were 15.0 and 6.5 years old, respectively. The mean duration of ERT for the patients with attenuated and severe type of MPS II were 5.5 and 6.5 years, respectively. The mean ages of sample collection of the patients with attenuated and severe type of MPS II were 21.5 and 13.0 years old, respectively. Among 4 and 4 patients of the attenuated and severe type of MPS II, 2 and 2 patients were positive for antibody against IDS. Two patients of the attenuated type of MPS II had not been determined the antibody titer and two patients of severe type of MPS II were reported negative for antibody production.

### 2.3 Approval by institutional Research Ethics Board

This study was approved by the Research Ethics Board of the National Center for Child Health and Development.

### 2.4 Sample preparation and analysis by LC-MS/MS

The preparation and analysis of urine samples by LC-MS/MS assay has been previously reported [11], [12]. In brief, an aliquot of urine samples (50 μL) was evaporated to dryness using a vacuum centrifuge (model CVE3100, EYELA, Tokyo, Japan). The residue was incubated with methanolic 3N-HCl (100 μL) at 65 °C for 75 min, followed by dryness with nitrogen. The residue was then dissolved in the mobile phase of UPLC (100 μL). An aliquot (0.5 μL) was injected into a Quattro Premier mass spectrometer equipped with an Acquity UPLC system (Waters Corporation, Milford, MA). Disaccharides were separated on an Atlantis T3 C18 column (1.7 μm, 2.1 × 50 mm, Waters Corporation) over 10 min at 0.1 mL/min as described in Supplementary Tables 1–3. The data were acquired by multiple reaction monitoring (MRM) using the specific transitions corresponding to HS, DS and CS, respectively. Before injection, all of the clinical samples were passed through a disposable filter (Fast Remover for Protein, 0.45 μm, GL Sciences, Tokyo, Japan) to minimize the ion suppression for measurement by endogenous contaminants.

### 2.5 Total GAG assay by 1,9-dimethylmethylene blue (DMB)-based colorimetric method

Total GAG was determined using the Blyscan kit (Blyscan Sulfated Glycosaminoglycan Assay), a colorimetric procedure using 1,9-dimethylmethylene blue (DMB) (Biocolor Ltd., Northern Ireland, UK) [12].

### 2.6 Creatinine assay

Urinary creatinine concentrations were determined spectrophotometrically using the Creatinine Colorimetric Assay kit (Cayman Chemicals, Ann Arbor, MI) [15].

### 2.7 Statistical analysis

Data were expressed as either mean ± SD or mean ± SEM, where indicated. Mean values of the two groups were compared using a Student's *t*-test and the difference was considered statistically significant when *P* < 0.05.

## 3 Results and discussion

### 3.1 Validation of methodology

To exclude the possibility that the co-migration of endogenous materials with HS might take place, we first examined whether the spiked HS could be quantitatively recovered in our experimental procedure. As shown in Table 1, the known amount of HS (i.e. 10 μg/mL) spiked into the urine was detected at 8.2 ± 0.7 μg/mL (mean ± SD), demonstrating that 82% of HS was recovered in this analytical procedure. To further examine the variance of measurement of HS, we determined the value of CV (%) of intraday and interday measurement. As shown in Table 2, the values for intraday and interday CV of HS were 8.0–28.1 and 4.8–12.7%, respectively. Thus, these results demonstrated that the levels of HS derived from urinary GAGs from MPS II patients were measured quatitatively. For DS and CS, their recoveries from the urine and intraday CV values resulted similarly compared to those for HS.

### 3.2 Levels of HS and DS in the urinary GAGs of MPS II patients

**Fig. 1:**
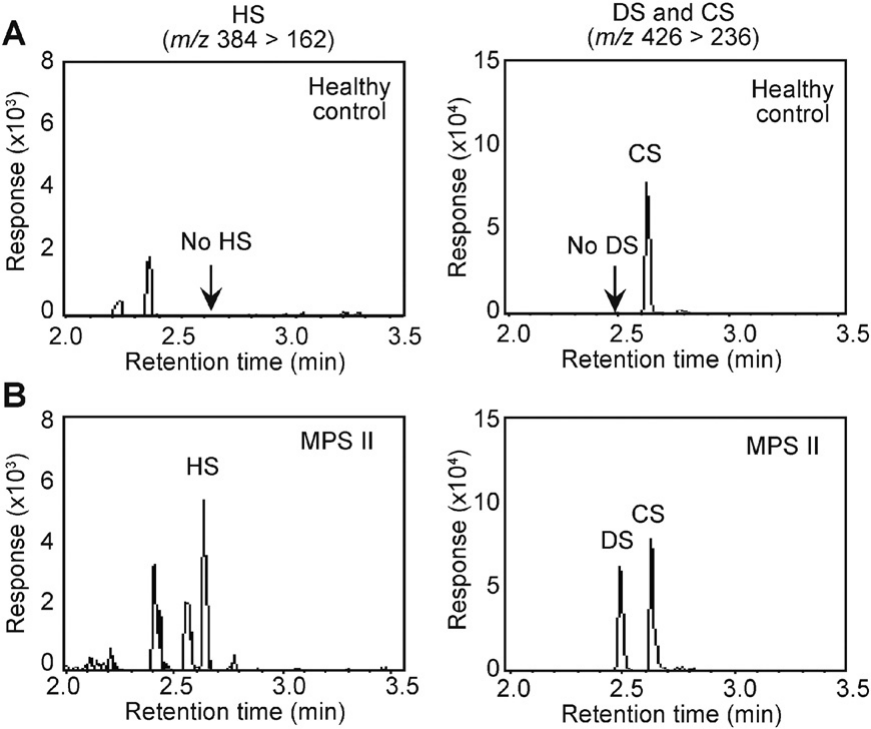
Representative chromatograms of the HS, DS and CS in the urinary GAGs determined using LC-MS/MS. Representative chromatograms of HS (left, *m/z* 384 > 162) and DS and CS (right, *m/z* 426 > 236) from a healthy human subject (A) and a 13-year-old MPS-II patient (attenuated type, B) were shown.

Fig. 1 shows the representative chromatograms for HS, DS and CS in the urinary GAGs in the humans. As reported previously, the healthy human urine only contained detectable amount of CS, whereas both DS and HS were undetectable (Fig. 1A). The detection limit (S/N = 3) for HS, DS and CS were 1 μg/mL for these GAGs; these values were calculated by a peak height of known amount of GAGs divided by an averaged range of baseline (Supplementary Table 4). In contrast to the healthy control, we readily detected the accumulating HS and DS in the urine from the MPS II patient (Fig. 1B).

The summary of the concentrations of HS and DS in the urinary GAGs of MPS II-affected individuals and their associated clinical information were provided in Table 3. On average, the ages at the sample collection of the patients were 21.5 and 13.0 years old for the attenuated and severe type of MPS II, respectively (Table 3, Table 4). In this study, all of the MPS II-affected individuals showed higher concentrations of HS + DS + CS compared to those of the healthy controls.

Next, we compared the concentrations of HS and DS within the severe and attenuated type of MPS II to understand whether antibody production may affect the concentrations of HS and DS. In the severe type of MPS II-affected individuals, the concentrations of HS of 2 antibody-positive patients were 151.9 and 144.5 mg/g Cr, whereas those of 2 antibody-negative patients were 64.4 and 79.8 mg/g Cr, respectively, showing clearly that the concentration of HS was correlated with the production of antibody. In contrast, the concentrations of DS of these antibody-positive patients were 112.9 and 73.4 mg/g Cr, whereas those of antibody-negative patients were 96.7 and 105.6 mg/g Cr, respectively. Thus, the concentration of DS of the severe type was not affected by the accumulation of antibody. Similarly, among the patients with attenuated type of MPS II, the patient who examined 2 months after the ERT initiation showed the highest concentrations of HS and DS by LC-MS/MS (HS, 181.1; DS, 171.2 mg/g Cr). Compared to these values, the concentrations of HS and DS of 2 patients with antibody-positives were lower than this patient, but higher than a patient who received ERT and HSCT (HS, 24.8; DS, 42.2 mg/g Cr).

Then, we compared the concentrations of HS and DS of the attenuated and severe type of MPS II-affected individuals whether the severity of disease may affect the concentrations of HS and DS. When 2 patients with the attenuated type and 2 patients with the severe type of MPS II, all of them were antibody-positive, were compared, the concentrations of HS in the severe type of MPS II (151.9 and 144.5 mg/g Cr) were higher than those of the attenuated type (56.2 and 83.7 mg/g Cr). Similarly, the comparison between 2 patients of the severe type without antibody and 1 patient of the attenuated type with ERT and HSCT, whose titer was not examined, revealed that the concentrations of HS and DS by LC-MS/MS in the severe type of MPS II-affected individuals were elevated. Although the titer of the patient of the attenuated type with ERT and HSCT was not examined, the concentrations of HS and DS of this patient were also below those with severe type of MPS II of antibody-positive group.

### 3.3 Effect of antibody production

During ERT, it is occasionally observed the accumulation of antibody against exogenously infused IDS protein [16]. In this previous study, the reduction in urinary GAGs by ERT was attenuated in antibody-positive patients. Consistent with this observation, the higher accumulation of HS in the urine from antibody-positive patients was also observed in this study (Table 3). Based on our data, the continuous monitoring of the concentration of HS in the urine may be effective for the estimation of antibody accumulation. The previous studies have shown the efficacy of ERT have been followed for 1.5 years [10], [14] and 3 years [11], respectively. Within our examination, a patient of the severe type of MPS II who received ERT for 3 years 10 months showed an accumulation of antibody (Table 3). Thus, the continuous monitoring of the concentrations of HS and DS after ERT initiation was considered to be informative due to the follow-up of ERT and estimation of antibody accumulation for both types of MPS II.

### 3.4 Clinically available specific biomarkers to identify the severe type of MPS II

There is a potential demand for the discrimination of the disease subtype of MPS II through a non-invasive manner. It is generally accepted that the urinary GAGs decreases over the age [17]. This, at least in part, is affected by the concentration of creatinine, because it increases over age due to the increasing mass of muscle [14]. In the urine of the patients of MPS II with the pathology of the central nerve system, Nielsen et al. reported the concentration of tri-sulfated tetrasaccharide was elevated [18]. Importantly, a recent report has shown the selective accumulation of total GAGs in the CSF is correlated with the impairment of cognitive function in MPS II in humans [8]. Notably, the LC-MS/MS-based methodology used this study has been applied to the CSF obtained from patients of MPS I [9]. Thus, one of promising method to discriminate the disease subtypes of MPS II is to apply this methodology to the CSF of MPS II patients. Alternatively, a better biomarker that selectively accumulates in the non-invasive clinical specimens, such as urine and saliva, needs to be further explored.

### 3.5 Assay for keratin sulfate (KS)

Among various GAGs in MPSs, KS is specifically elevated in MPS IV. As a biochemical marker for diagnosis, KS and CS are accumulating in MPS IVA [19], whereas a selective accumulation of KS in MPS IVB has been reported [20]. The concentration of KS has been determined with the pretreatment of KS in the urinary GAGs with keratanase II followed by measurement with isotope dilution technique using LC-MS/MS [21], [22]. From clinical point of view, the development of KS assay which can simultaneously determine the concentrations of HS, DS and CS will be awaited.

In conclusion, we presented the levels of HS and DS in MPS II-affected patients using LC-MS/MS. Both were readily detectable in the patients, but not in the healthy subjects. The disease subtypes were identified using this LC-MS/MS method when the detailed history of patients was provided. A potential biomarker to discriminate the attenuated and severe type of MPS II in the non-invasive clinical specimen still remains to be explored.

## Figures and Tables

**Table 1:** Recovery of the spiked GAGs from the urine of a healthy subject.

Additive	Added concentration (μg/mL)	Recovered concentration (μg/mL)	Recovery^a^ (%)	Replicate *n*
HS	10	8.2 ± 0.7	82 ± 7	5
DS	10	8.9 ± 0.5	89 ± 5	5
CS^b^	10	16.6 ± 4.7	166 ± 47	3

Data were expressed as mean ± SD in at least triplicate determination.

a Recovery is defined as the concentration of recovered GAGs/the concentration of added GAGs.

b Endogenous concentration of CS was 60.0 μg/mL.

**Table 2:** The values of intra- and interday CV (%) for the concentrations of HS, DS and CS of the MPS II-affected individuals using LC-MS/MS.

MPS II	Sample collection (y)	Intraday CV (%)			Interday CV (%)
HS (*n* = 5)	DS (*n* = 5)	CS (*n* = 5)	HS (*n* = 3)
Attenuated type	23	4.8	1.0	3.9	12.9
	10	12.7	1.5	2.2	28.1
	13	5.0	2.3	3.3	14.3
	40	7.1	2.3	0.3	9.7
Severe type	17	7.6	1.5	3.0	8.0
	10	9.9	2.4	3.1	15.5
	14	9.9	3.4	5.2	13.4
	11	5.7	1.3	3.8	9.8

CV, coefficient of variance.

**Table 3:** The concentrations of HS, DS, CS and total GAGs of the patients with the attenuated and severe type of MPS II. Urinary GAGs were pretreated with 3 M HCl in methanol at 65 °C for 75 min followed by the quantification using LC-MS/MS. Units are in mg/g Cr.

Classification	Disease subtype	ERT (Y/N)	HSCT (Y/N)	ERT initiation	Duration of ERT	Antibody production	Sample collection	DMB	LC-MS/MS			
Total GAG	HS + DS + CS	HS	DS	CS
		(y)	(y/m)		(y)	(mg/g Cr)				
MPS II	Attenuated	Yes	No	39	0/2	Not determined	40	144.9	458.4	181.1	171.2	106.1
	Attenuated	Yes	No	2	7/5	Positive	10	88.9	239.2	56.2	79.1	103.9
	Attenuated	Yes	No	4	7/5	Positive	13	70.2	370.7	83.7	125.7	161.4
	Attenuated	Yes	Yes/CBT	15	7/0	Not determined	23	38.3	103.3	24.8	42.2	36.4
	Severe	Yes	No	6	3/10	Positive	10	86.6	374.9	151.9	112.9	110.0
	Severe	Yes	No	6	7/6	Positive	14	69.7	330.3	144.5	73.4	112.4
	Severe	Yes	No	10	7/0	Negative	17	124.6	268.3	64.4	96.7	107.2
	Severe	Yes	No	4	7/0	Negative	11	63.0	309.8	79.8	105.6	124.5
Healthy controls	N/A	N/A	N/A	N/A	N/A	N/A	43	69.6	33.4	ND	ND	33.4
	N/A	N/A	N/A	N/A	N/A	N/A	11	34.1	36.3	ND	ND	36.3
	N/A	N/A	N/A	N/A	N/A	N/A	41	45.4	24.3	ND	ND	24.3
	N/A	N/A	N/A	N/A	N/A	N/A	8	67.7	51.8	ND	ND	51.8

CBT, cord blood transplant; ND, not detected; N/A, not applicable.

**Table 4:** Statistical results of the levels of urinary GAGs of the patients with the attenuated and severe types of MPS II in this study.

MPS II subtype	*n*		ERT initiation	Duration of ERT	Sample collection	DMB	LC-MS/MS			
Total GAG	HS + DS + CS	HS	DS	CS
(y)	(y)	(y)	(mg/g Cr)				
Attenuated type	4	mean	15.0	5.5	21.5	85.6	292.9	86.4	104.5	101.9
		SEM	8.5	1.8	6.8	22.3	77.6	33.8	28.0	25.6
Severe type	4	mean	6.5	6.5	13.0	86.0	320.8	110.2	97.1	113.5
		SEM	1.3	0.9	1.6	13.8	22.1	22.2	8.6	3.8
		Student's *t*	0.180	0.323	0.133	0.494	0.371	0.289	0.404	0.335

SEM, standard error of the mean.

## Appendix A Supplementary data

Supplementary tables. Image 1

## References

[1] Muenzer J. (2004). The mucopolysaccharidoses: a heterogeneous group of disorders with variable pediatric presentations. J. Pediatr..

[2] Wraith J.E., Clarke L.A., Beck M., Kolodny E.H., Pastores G.M., Muenzer J., Rapoport D.M., Berger K.I., Swiedler S.J., Kakkis E.D., Braakman T., Chadbourne E., Walton-Bowen K., Cox G.F. (2004). Enzyme replacement therapy for mucopolysaccharidosis I: a randomized, double-blinded, placebo-controlled, multinational study of recombinant human alpha-L-iduronidase (laronidase). J. Pediatr..

[3] Muenzer J., Gucsavas-Calikoglu M., McCandless S.E., Schuetz T.J., Kimura A. (2007). A phase I/II clinical trial of enzyme replacement therapy in mucopolysaccharidosis II (Hunter syndrome). Mol. Genet. Metab..

[4] Muenzer J., Hendriksz C.J., Fan Z., Vijayaraghavan S., Perry V., Santra S., Solanki G.A., Mascelli M.A., Pan L., Wang N., Sciarappa K., Barbier A.J. (2015). A phase I/II study of intrathecal idursulfase-IT in children with severe mucopolysaccharidosis II. Genet. Med..

[5] Boado R.J., Ka-Wai Hui E., Zhiqiang Lu J., Pardridge W.M. (2014). Insulin receptor antibody-iduronate 2-sulfatase fusion protein: pharmacokinetics, anti-drug antibody, and safety pharmacology in Rhesus monkeys. Biotechnol. Bioeng..

[6] Muenzer J., Wraith J.E., Clarke L.A. (2009). Mucopolysaccharidosis I: management and treatment guidelines. Pediatrics.

[7] Peters C., Balthazor M., Shapiro E.G., King R.J., Kollman C., Hegland J.D., Henslee-Downey J., Trigg M.E., Cowan M.J., Sanders J., Bunin N., Weinstein H., Lenarsky C., Falk P., Harris R., Bowen T., Williams T.E., Grayson G.H., Warkentin P., Sender L., Cool V.A., Crittenden M., Packman S., Kaplan P., Lockman L.A., Anderson J., Krivit W., Dusenbery K., Wagner J. (1996). Outcome of unrelated donor bone marrow transplantation in 40 children with Hurler syndrome. Blood.

[8] Hendriksz C.J., Muenzer J., Vanderver A., Davis J.M., Burton B.K., Mendelsohn N.J., Wang N., Pan L., Pano A., Barbier A.J. (2015). Levels of glycosaminoglycans in the cerebrospinal fluid of healthy young adults, surrogate-normal children, and Hunter syndrome patients with and without cognitive impairment. Mol. Genet. Metab. Rep..

[9] Zhang H., Young S.P., Auray-Blais C., Orchard P.J., Tolar J., Millington D.S. (2011). Analysis of glycosaminoglycans in cerebrospinal fluid from patients with mucopolysaccharidoses by isotope-dilution ultra-performance liquid chromatography-tandem mass spectrometry. Clin. Chem..

[10] Auray-Blais C., Bherer P., Gagnon R., Young S.P., Zhang H.H., An Y., Clarke J.T., Millington D.S. (2011). Efficient analysis of urinary glycosaminoglycans by LC-MS/MS in mucopolysaccharidoses type I, II and VI. Mol. Genet. Metab..

[11] Chuang C.K., Lin H.Y., Wang T.J., Tsai C.C., Liu H.L., Lin S.P. (2014). A modified liquid chromatography/tandem mass spectrometry method for predominant disaccharide units of urinary glycosaminoglycans in patients with mucopolysaccharidoses. Orphanet J. Rare Dis..

[12] Zhang H., Wood T., Young S.P., Millington D.S. (2015). A straightforward, quantitative ultra-performance liquid chromatography-tandem mass spectrometric method for heparan sulfate, dermatan sulfate and chondroitin sulfate in urine: an improved clinical screening test for the mucopolysaccharidoses. Mol. Genet. Metab..

[13] Trim P.J., Hopwood J.J., Snel M.F. (2015). Butanolysis derivatization: improved sensitivity in LC-MS/MS quantitation of heparan sulfate in urine from mucopolysaccharidosis patients. Anal. Chem..

[14] Auray-Blais C., Lavoie P., Zhang H., Gagnon R., Clarke J.T., Maranda B., Young S.P., An Y., Millington D.S. (2012). An improved method for glycosaminoglycan analysis by LC-MS/MS of urine samples collected on filter paper. Clin. Chim. Acta.

[15] Husdan H., Rapoport A. (1968). Estimation of creatinine by the Jaffe reaction. A comparison of three methods. Clin. Chem..

[16] Muenzer J., Wraith J.E., Beck M., Giugliani R., Harmatz P., Eng C.M., Vellodi A., Martin R., Ramaswami U., Gucsavas-Calikoglu M., Vijayaraghavan S., Wendt S., Puga A.C., Ulbrich B., Shinawi M., Cleary M., Piper D., Conway A.M., Kimura A. (2006). A phase II/III clinical study of enzyme replacement therapy with idursulfase in mucopolysaccharidosis II (Hunter syndrome). Genet. Med..

[17] Langereis E.J., Wagemans T., Kulik W., Lefeber D.J., van Lenthe H., Oussoren E., van der Ploeg A.T., Ruijter G.J., Wevers R.A., Wijburg F.A., van Vlies N. (2015). A multiplex assay for the diagnosis of mucopolysaccharidoses and mucolipidoses. PLoS One.

[18] Nielsen T.C., Rozek T., Hopwood J.J., Fuller M. (2010). Determination of urinary oligosaccharides by high-performance liquid chromatography/electrospray ionization-tandem mass spectrometry: application to Hunter syndrome. Anal. Biochem..

[19] Shimada T., Tomatsu S., Yasuda E., Mason R.W., Mackenzie W.G., Shibata Y., Kubaski F., Giugliani R., Yamaguchi S., Suzuki Y., Orii K., Orii T. (2014). Chondroitin 6-Sulfate as a Novel Biomarker for Mucopolysaccharidosis IVA and VII JIMD Rep 16.

[20] van der Horst G.T., Kleijer W.J., Hoogeveen A.T., Huijmans J.G., Blom W., van Diggelen O.P. (1983). Morquio B syndrome: a primary defect in beta-galactosidase. Am. J. Med. Genet..

[21] Martell L.A., Cunico R.L., Ohh J., Fulkerson W., Furneaux R., Foehr E.D. (2011). Validation of an LC-MS/MS assay for detecting relevant disaccharides from keratan sulfate as a biomarker for Morquio A syndrome. Bioanalysis.

[22] Auray-Blais C., Lavoie P., Maranda B., Boutin M. (2016). Evaluation of urinary keratan sulfate disaccharides in MPS IVA patients using UPLC-MS/MS. Bioanalysis.

