# Supplementary material for: The levels of urinary glycosaminoglycans of patients with attenuated and severe type of mucopolysaccharidosis II determined by liquid chromatography-tandem mass spectrometry
Source: Mol Genet Metab Rep. 2016 Apr 22;7:87–91. doi: 10.1016/j.ymgmr.2016.03.009 (PMC4908047; doi:10.1016/j.ymgmr.2016.03.009)
Supplement: Supplementary file 1 — Supplementary tables. [file mmc1.pdf]

Supplementary Table 1

UPLC method for the quantitation and fragmentation study of HS, DS and CS in the urinary GAGs in humans.

Column	Waters Atlantis T3 50 mm
	Internal diameter: 2.1 mm
	Particle diameter: 3 μm
Column temperature	45 °C
Weak wash solvent	MeOH/H_2O
Strong wash solvent	MeOH/ACN/IPA/0.1 % formic acid in H_2O
Mobile phase A	0.1 % formic acid/5 mM ammonium acetate
Mobile phase B	0.1 % formic acid in MeOH
Gradient (%B)	0 - 1.5 min: 1 %
	1.5 - 5 min: 1 - 90 %
	5 - 6 min: 90 %
	6.01 - 8.0 min: 1%
Flow rate	0.4 mL/min
Injection volume	0.5 μL
Injection mode	Direct injection
Autosampler temperature	4 °C

Supplementary Table 2

Instrument parameters for the fragmentation study of HS, DS and CS in the urinary GAGs in humans using LC-MS/MS.

Interface	ESI
Polarity	Positive
Capillary voltage	4.5 kV
Source temperature	250 °C
Desolvation temperature	400 °C
Flow rate of cone gas	50 L/hr
Flow rate of desolvation gas	800 L/hr
Analyzing mode	MRM
Scan time	0.1 s
Data format	Centroid

Supplementary Table 3

Product ion, precursor ion and collision energy for the fragmentation study of HS, DS and CS in the urinary GAGs in humans using LC-MS/MS.

	Precursor ion (*m/z*)	Product ion (*m/z*)	Collision energy (V)
HS	384	162	-16
DS	426	236	-10
CS	426	236	-10

Supplementary Table 4

Limit of detection (LOD) for the GAG.

Species	LOD (μg/mL)
HS	1.0
DS	1.0
CS	1.0

[a]Limit of detection is defined as S/N=3.
